# Public Health Impact and Economic Costs of Volkswagen’s Lack of Compliance with the United States’ Emission Standards

**DOI:** 10.3390/ijerph13090891

**Published:** 2016-09-08

**Authors:** Lifang Hou, Kai Zhang, Moira A. Luthin, Andrea A. Baccarelli

**Affiliations:** 1 Department of Preventive Medicine and the Robert H. Lurie Comprehensive Cancer Center, Feinberg School of Medicine, Northwestern University, Chicago, IL 60611, USA; l-hou@northwestern.edu; 2 Department of Epidemiology, Human Genetics and Environmental Sciences, University of Texas School of Public Health, Houston, TX 77030, USA; 3 Department of Environmental Health, Harvard T.H. Chan School of Public Health, Boston, MA 02115, USA; moira.luthin@gmail.com; 4 Department of Environmental Health Sciences, Columbia University Mailman School of Public Health, New York, NY 10032, USA; andrea.baccarelli@columbia.edu

**Keywords:** nitrogen oxides, risk assessment, vehicle emissions

## Abstract

The U.S. Environmental Protection Agency (EPA) recently issued a notice of violation against Volkswagen (VW) for installing a defective device in certain models of diesel cars to circumvent emission tests for nitrogen oxides (NO_x_). We quantified the health and economic impacts of extra NO_x_ emissions attributable to non-compliant vehicles in the U.S. using the EPA’s Co-Benefits Risk Assessment model. We estimated that the total extra NO_x_ emitted over one year of operation would result in 5 to 50 premature deaths, 687 to 17,526 work days with restricted activity, and economic costs of $43,479,189 to $423,268,502, based on various assumptions regarding emission scenarios and risks. This study highlights the potential impacts of VW vehicles’ lack of compliance on the health and well-being of the U.S. population.

## 1. Introduction

On 18 September 2015, the United States (U.S.) Environmental Protection Agency (EPA) issued a notice of violation of the Clean Air Act to Volkswagen (VW) AG, Audi AG, and VW Group of America [1], sparking public uproar. Volkswagen illegally installed a defeat software in approximately 482,000 diesel-powered passenger cars in the U.S. since 2008. This software circumvents the U.S. EPA’s emission tests by turning off the nitrogen oxides (NO_x_) emission control system in real-world driving. Ambient air pollution was estimated to contribute to ~3.7 million deaths globally in 2012 [2]. Vehicles are a major source of air pollution in urban areas, and traffic-related air pollutants have been linked to many adverse health effects, including mortality, non-allergic respiratory morbidity, allergic illness and symptoms, cardiovascular morbidity, cancer, preterm birth, and decreased male fertility [3]. Measuring the health and economic impacts of air pollution typically consists of three key steps: assessing exposure, estimating health impact by linking exposure to selected exposure–response relationships from the literature, and converting that health impact into an equivalent dollar value [4]. Most studies on the impact of air pollution tend to be similar in these last two steps because they primarily use the same well-established exposure–response and economic data from the literature. Exposure to air pollution has generally been assigned using the interpolated values of monitoring data or predictions generated from dispersion models—e.g., the EPA’s Community Multi-scale Air Quality (CMAQ) Modeling System. Although air quality models like CMAQ provide exposure estimates at fine spatial and temporal resolutions, they are computationally intensive, especially at a large (e.g., state and national level) scale [5]. Reduced-form air quality models that are computationally efficient and easy to use have been developed and used in previous studies [5,6,7], and several health impact assessment tools relying on these reduced-form models have been provided by the EPA—e.g., the Co-Benefits Risk Assessment (COBRA) screening model. COBRA has been used in previous studies to evaluate the health impact of a technology or policy [5,8]. 

Despite speculation regarding the public health impact caused by VW’s lack of transparency, the expected toll on human health and related external economic costs remains unaddressed. The purpose of this research is to estimate the health and economic impact of Volkswagen’s non-compliance across the U.S. using the EPA’s COBRA screening tool.

## 2. Methods

The EPA determined that VW illegally installed software in certain diesel-powered passenger car models that, in real-world driving, bypass a built-in NO_x_ abatement system [1]. In the U.S., 482,000 cars equipped with 2.0-liter diesel engines were affected [1]. We used COBRA to estimate the numbers of premature deaths, health conditions, and lost work days attributable to the non-compliant vehicles and all related economic impacts [9]. COBRA is a peer-reviewed screening model developed for the EPA that estimates health and economic benefits or costs of different air quality regulations [9]. The model’s inputs include changes in the emissions of fine particulate matter (≤2.5 µm in diameter, PM_2.5_), sulfur dioxide (SO_2_), NO_x_, ammonia, and volatile organic compounds by source category at various spatial scales (e.g., county, state, and national levels). With NO_x_ emission changes provided by users, COBRA uses a reduced-form air quality model to derive changes of ambient PM concentrations due to these NO_x_ emission changes and then estimates health effects attributable to PM. This reduced-form air quality model is called the Source-Receptor (S–R) Matrix. The S–R matrix provides transfer coefficients between emissions and county-level PM_2.5_ concentrations in the U.S. via a sector-based Gaussian dispersion model that accounts for wet and dry deposition and first-order chemical transformations in the atmosphere, and uses meteorological inputs calculated based on weather observations in 1990. Initial predictions of PM_2.5_ concentrations have been calibrated using the EPA’s ambient monitoring data. The S–R matrix has been used previously in the EPA’s regulatory impact analysis [7] and peer-reviewed publications [6]. COBRA uses predicted PM_2.5_ concentrations at the county level as a proxy of personal exposure to PM_2.5_ for people living in those counties, and estimates the health impact by combining these exposures with exposure–response relationships for each selected health outcome recommended by the EPA based on the literature. Finally, COBRA converts health impacts into an economic impact using an approach similar to that used in the EPA’s regulatory impact analysis.

## 3. Results

According to the EPA, NO_x_ emissions from non-compliant cars were 10 to 40 times higher than the EPA’s current Tier 2 vehicle emission standard (0.07 grams/mile) [10,11]. We defined these boundaries as best-case (10×) and worst-case (40×) scenarios, and the midpoint (25×) as intermediate (Table 1). According to state-reported Highway Performance and Monitoring System data, in 2013, the annual mileage of the average passenger car in the U.S. was 11,244 miles [12]. In the absence of vehicle-specific mileage data, we assume this value for all affected cars in order to calculate total additional emissions. Thus, over one year of operation, the 482,000 non-compliant cars would emit a total of 3414 to 14,796 metric tons of extra NO_x_ in the best- and worst-case scenarios, respectively.

We estimated that across the different emission scenarios the total extra NO_x_ emitted over one year of operation by the 482,000 non-compliant cars would result in 5 to 12 premature deaths using the EPA’s lower-mortality assumption, 22 to 50 premature deaths using the EPA’s higher-mortality assumption, 247 to 1061 episodes of respiratory symptoms, 3 to 14 hospital admissions for cardiorespiratory causes, and 3 to 13 emergency visits for asthma. These health effects would result in a loss of 687 to 2946 work days, and 4085 to 17,526 work days with restricted activity (Table 1). The associated economic costs range from $43,479,189 to $423,268,502, based on various assumptions regarding emission scenarios and risks (Table 2). The majority of the estimated costs relate to the economic impact of premature deaths (Table 2).

## 4. Discussion

Our study suggests that VW vehicles’ lack of compliance had impacts on the health and well-being of the U.S. population. This assessment has limitations. First, we could not break down the affected cars by model, year of manufacture, or geographic area due to lack of data. Therefore, we produced estimates over one year of operation for all 482,000 affected cars. These estimates should be roughly equivalent to current annual estimates, as non-compliant cars were released starting in 2008 and, due to the low expected attrition of relatively new vehicles, most of them likely remain active. Although these estimates of health impact and external costs are much smaller than those for the EPA’s 1990 Clean Air Act Amendments [15] (e.g., 160,000 prevented adult deaths and 1200 billion dollars in 2010) these estimates are non-negligible considering that this analysis focuses only on additional NO_x_ emissions attributable to affected VW cars. Second, due to lack of data we did not include those light duty diesel cars equipped with 3.0-liter diesel engines that also installed a defeat device to circumvent emission tests for NO_x_ [16], nor did we include those diesel and gas cars with underreported carbon dioxide emissions [17]. Third, large uncertainties exist in most of these calculations, including estimates of emissions and concentrations, exposure–response relationships, and monetization of health impact. Finally, this analysis inherits all limitations inherent in the COBRA model, including overlooking the complex chemical reactions and transport of NO_x_ in the air or for varying weather conditions by geographic areas, its indirect estimation of NO_x_ impacts through NO_x_-derived fine particles, and lack of consideration of ozone contributed by NO_x_ emissions through complicated photochemical reactions (increased NO_x_ emissions can decrease ozone levels near roads, while ozone concentrations downwind increase) [18]. Future investigations are required to account for the geographical distribution of affected cars, varying weather conditions, and ozone attributable to extra NO_x_ emissions. More accurate emission estimates can be calculated based on more detailed information on the affected VW cars (e.g., actual emission rates per category and model year, annual traveled distance per category, weather conditions). Predictions of air pollutant concentrations can be derived from regional- or global-scale air quality models that simulate chemical reactions related to NO_x_ emissions (e.g., CMAQ). 

## 5. Conclusions

This study shows that VW vehicles’ lack of compliance potentially had impacts on the health and well-being of the U.S. population. When considering VW’s alleged misdeeds, the consequences to public health and ensuing economic impact must be considered in further refined studies. 

## Figures and Tables

**Table 1:** Total emissions and estimated annual premature deaths, hospital admissions, and diseases attributable to Volkswagen vehicles’ lack of compliance with EPA standards in the U.S.

Emissions and Health Outcomes	Best-Case Scenario	Midpoint	Worst-Case Scenario
Emissions			
Assumed NO_x_ emissions against EPA’s current Tier 2 vehicle emission standard (0.07 grams per mile) ^a^	10×	25×	40×
Metric tons of extra NO_x_ released annually by non-compliant vehicles	3414	9105	14,796
Mortality			
Premature deaths in adults, lower-risk assumption ^b^	5	13	22
Premature deaths in adults, higher-risk assumption ^b^	12	31	50
Diseases and symptoms			
Asthma exacerbations	154	407	660
Acute bronchitis	8	21	34
All respiratory symptoms	247	654	1061
Hospital admissions			
Hospital admissions related to respiratory and cardiovascular diseases	3	9	14
Asthma emergency room visits	3	8	13
Impact on work productivity			
Days of work lost	687	1816	2946
Days with minor restrictions in activity	4085	10,805	17,526

^a^ Best-case, midpoint, and worst-case scenarios assume NO_x_ emissions released from VW affected cars were respectively 10, 25, or 40 times higher on average than the EPA standard of 0.07 grams per mile; ^b^ The EPA Co-Benefits Risk Assessment (COBRA) model generates low and high boundaries of estimates for premature deaths and heart attacks in adults based on high and low reports of risk from different reference studies.

**Table 2:** Estimated annual economic costs (2010 US dollars) of premature deaths, hospital admissions, and diseases attributable to Volkswagen vehicles’ lack of compliance with EPA standards in the United States ^a^.

Health Outcomes	Best-Case Scenario	Midpoint	Worst-Case Scenario
Mortality			
Premature deaths in adults, lower risk assumption ^b^	$42,777,243	$113,441,872	$184,106,754
Premature deaths in adults, higher risk assumption ^b^	$97,013,943	$257,278,035	$417,543,579
Diseases and symptoms			
Asthma exacerbations	$8,804	$23,302	$37,800
Acute bronchitis	$3,804	$10,066	$16,329
All respiratory symptoms	$6,918	$18,307	$1,061
Hospital admissions			
Hospital admissions related to respiratory and cardiovascular diseases	$110,216	$292,655	$475,095
Asthma emergency room visits	$1,273	$3,382	$5,491
Impact on work productivity			
Days of work lost	$109,665	$290,091	$470,517
Days with minor restrictions in activity	$276,577	$731,636	$1,186,696
Total economic losses, lower-risk assumptions ^b^	$43,479,189	$115,301,466	$187,124,000
Total economic losses, higher-risk assumptions ^b^	$98,344,726	$260,805,876	$423,268,502

^a^ A 3% discount rate was used in estimating economic value; ^b^ The EPA COBRA model generates low and high boundaries of estimates for premature deaths in adults and other outcomes based on high and low reports of risk from different reference studies (lower and higher risk assumptions use the concentration–response functions from two studies: Krewski et al. (2009) [13] and Lepeule et al. (2012) [14], respectively). This table shows selected endpoints from the COBRA model’s results. Thus, the estimates of total economic losses are larger than the sum of economic costs for each endpoint shown in this table.

## Abbreviations

The following abbreviations are used in this manuscript:

COBRA	Co–Benefits Risk Assessment
EPA	Environmental Protection Agency
VW	Volkswagen

## References

[1] U.S. Environmental Protection Agency (EPA) EPA, California Notify Volkswagen of Clean Air Act Violations/Carmaker Allegedly Used Software That Circumvents Emissions Testing for Certain Air Pollutants, 2015. http://yosemite.epa.gov/opa/admpress.nsf/6424ac1caa800aab85257359003f5337/dfc8e33b5ab162b985257ec40057813b!OpenDocument.

[2] World Health Organization (2012). Global Health Observatory. http://apps.who.int/gho/data/node.main.156?lang=en.

[3] Health Effects Institute Traffic-Related Air Pollution: A Critical Review of the Literature on Emissions, Exposure, and Health Effects, 2010. http://pubs.healtheffects.org/getfile.php?u=553.

[4] U.S. Environmental Protection Agency (EPA) The Benefits and Costs of the Clean Air Act from 1990 to 2020, 2010. https://www.epa.gov/sites/production/files/2015-07/documents/fullreport_rev_a.pdf.

[5] Bridges A., Felder F.A., McKelvey K., Niyogi I. (2015). Uncertainty in energy planning: Estimating the health impacts of air pollution from fossil fuel electricity generation. Energy Res. Soc. Sci..

[6] Levy J.I., Wilson A.M., Zwack L.M. (2007). Quantifying the Efficiency and Equity Implications of Power Plant Air Pollution Control Strategies in the United States. Environ. Health Perspect..

[7] U.S. Environmental Protection Agency (EPA) (1999). Regulatory Impact Analysis—Control of Air Pollution from New Motor Vehicles: Tier 2 Motor Vehicle Emissions Standards and Gasoline Sulfur Control Requirements.

[8] McCubbin D., Sovacool B.K. (2013). Quantifying the health and environmental benefits of wind power to natural gas. Energy Policy.

[9] U.S. Environmental Protection Agency (EPA) User’s Manual for the Co-Benefits Risk Assessment (COBRA) Screening Model (version 2.7), 2015. http://www3.epa.gov/statelocalclimate/documents/pdf/COBRA_manual.pdf.

[10] U.S. Environmental Protection Agency (EPA) Tier 2 Exhaust Emission Standards and Implementation Schedule, 2012. http://www3.epa.gov/otaq/standards/light-duty/tier2stds.htm.

[11] U.S. Environmental Protection Agency (EPA) Notice of Violation, 2015. https://www.epa.gov/sites/production/files/2015-10/documents/vw-nov-caa-09-18-15.pdf.

[12] U.S. Federal Highway Administration Highway Statistics 2013, 2015. http://www.fhwa.dot.gov/policyinformation/statistics/2013/vm1.cfm.

[13] Krewski D., Jerrett M., Burnett R.T., Ma R., Hughes E., Shi Y., Turner M.C., Pope C.A., Thurston G., Calle E.E. (2009). Extended follow-up and spatial analysis of the American Cancer Society study linking particulate air pollution and mortality. Res. Rep. Health Eff. Inst..

[14] Lepeule J., Laden F., Dockery D., Schwartz J. (2012). Chronic exposure to fine particles and mortality: An extended follow-up of the Harvard Six Cities study from 1974 to 2009. Environ. Health Perspect..

[15] U.S. Environmental Protection Agency (EPA) Health and Welfare Benefits Analyses to Support the Second Section 812 Benefit-Cost Analysis of the Clean Air Act, 2011. https://www.epa.gov/sites/production/files/2015-07/documents/benefitsfullreport.pdf.

[16] U.S. Environmental Protection Agency (EPA) EPA, California Notify Volkswagen of Additional Clean Air Act Violations, 2015d. https://www.epa.gov/sites/production/files/2015-11/documents/vw-nov-2015-11-02.pdf.

[17] Volkswagenag (VW) AG Clarification Moving Forward: Internal Investigations at Volkswagen Identify Irregularities in CO_2_ Levels, 2015. http://www.volkswagenag.com/content/vwcorp/info_center/en/news/2015/11/internen_untersuchungen.html.

[18] National Research Council Estimating Mortality Risk Reduction and Economic Benefits from Controlling Ozone Air Pollution, 2008. http://www.nap.edu/read/12198.

